# Prostaglandin E_3_ attenuates macrophage‐associated inflammation and prostate tumour growth by modulating polarization

**DOI:** 10.1111/jcmm.16570

**Published:** 2021-05-13

**Authors:** Jing Cui, Kai Shan, Qin Yang, Yumin Qi, Hongyan Qu, Jiaqi Li, Rong Wang, Lingling Jia, Wei Chen, Ninghan Feng, Yong Q. Chen

**Affiliations:** ^1^ Wuxi School of Medicine Jiangnan University Wuxi China; ^2^ School of Food Science and Technology Jiangnan University Wuxi China; ^3^ Department of Urology Wuxi No. 2 People’s Hospital Wuxi China

**Keywords:** macrophage polarization, prostaglandin E_3_, protein kinase A, tumour‐associated macrophage

## Abstract

Alternative polarization of macrophages regulates multiple biological processes. While M1‐polarized macrophages generally mediate rapid immune responses, M2‐polarized macrophages induce chronic and mild immune responses. In either case, polyunsaturated fatty acid (PUFA)‐derived lipid mediators act as both products and regulators of macrophages. Prostaglandin E_3_ (PGE_3_) is an eicosanoid derived from eicosapentaenoic acid, which is converted by cyclooxygenase, followed by prostaglandin E synthase successively. We found that PGE_3_ played an anti‐inflammatory role by inhibiting LPS and interferon‐γ‐induced M1 polarization and promoting interleukin‐4‐mediated M2 polarization (M2a). Further, we found that although PGE_3_ had no direct effect on the growth of prostate cancer cells in vitro, PGE_3_ could inhibit prostate cancer in vivo in a nude mouse model of neoplasia. Notably, we found that PGE_3_ significantly inhibited prostate cancer cell growth in a cancer cell‐macrophage co‐culture system. Experimental results showed that PGE_3_ inhibited the polarization of tumour‐associated M2 macrophages (TAM), consequently producing indirect anti‐tumour activity. Mechanistically, we identified that PGE_3_ regulated the expression and activation of protein kinase A, which is critical for macrophage polarization. In summary, this study indicates that PGE_3_ can selectively promote M2a polarization, while inhibiting M1 and TAM polarization, thus exerting an anti‐inflammatory effect and anti‐tumour effect in prostate cancer.

## 1 INTRODUCTION

Macrophages can be activated in response to diverse stimuli and have distinct functional subsets resulting from distinct phenotypic polarization.^1^ According to the type‐1/type‐2 cell polarization theory,^2^, ^3^ phenotypically polarized macrophages are defined as one of two primary activation states, named as classically activated (M1) and alternatively activated (M2) macrophages. Classical M1 macrophages, which can be induced by lipopolysaccharide (LPS) and interferon (IFN)‐γ, exert pro‐inflammatory functions by secreting inflammatory factors. In anti‐infective immunity, M1 macrophages participate in the recognition and clearance of pathogens and can further activate cellular immunity. In the early stages of tumour development, M1 macrophages can also help activate immune surveillance to clear mutated cells. However, acute inflammation associated with M1 macrophages can also cause severe inflammatory damage, including organ failure induced by cytokine storms and accumulation of mutations through repeated injury. M2‐polarized macrophages can be divided into the following subtypes: M2a, M2b, M2c and M2d. Although these M2 subtypes share some markers (eg CD206), their activation and functions are different. M2a macrophages, which are induced by interleukin (IL)‐4 and/or IL‐13, are the most widely studied M2 subtype and attenuate the immune response by secreting IL‐10, transforming growth factor beta (TGF‐β), and C‐C motif chemokine ligand 17 (CCL17). The functions of M2b and M2c macrophages are similar to those of M2a macrophages but have distinct routes of polarization. M2d macrophages are also known as tumour‐associated macrophages (TAM) and reside in tumour microenvironments. Monocytes can be stimulated by secreted factors (eg chemokines, cytokines and growth factors) from cancer and non‐malignant cells in the tumour microenvironment to adopt a TAM phenotype. When activated, TAMs exhibit pro‐tumorigenic effects.

Prostaglandins (PGs) are a group of diverse polyunsaturated fatty acid‐derived bioactive lipids synthesized by cyclooxygenase (COX). In the synthesis of PGE_2_, arachidonic acid (AA) is first converted to prostaglandin H_2_ by the enzymes COX‐1 and COX‐2, and then cell‐specific prostaglandin synthases convert prostaglandin H_2_ into various prostaglandins, including PGI_2_, PGF_2α_, PGD_2_ and PGE_2_. Prostaglandins E_2_ and E_3_ (PGE_2_ and PGE_3_) have received the most attention for their roles in modulating inflammation.^4^, ^5^, ^6^ PGE_2_ has been well‐characterized and is known to play important roles in the regulation and activity of T lymphocytes.^7^, ^8^, ^9^ It primarily acts through four G protein‐coupled receptor subtypes of EP receptors, EP1, EP2, EP3 and EP4.^10^, ^11^ In addition, recent studies indicated that PGE_2_ can promote M2 polarization of macrophages in vivo through the EP4 receptor and its downstream cyclic AMP (cAMP)‐protein kinase A (PKA) pathway, which activates cAMP‐responsive element‐binding protein (CREB) and Kruppel‐like factor 4.^12^ PGE_2_ and PGE_3_ share the same metabolic pathway and have high structural similarity. However, unlike AA‐derived PGE_2_, eicosapentaenoic acid (EPA)‐derived PGE_3_ has not been widely studied. PGE_3_ is widely presumed to be anti‐inflammatory and anti‐neoplastic. It is thought that PGE_3_ can antagonize PGE_2_ to yield anti‐tumour or anti‐inflammatory effects, but there is a lack of supporting evidence.^13^ Moreover, there are few reports of PGE_3_ modulation of macrophage phenotypes. Existing studies suggest that PGE_3_ shares the same EP receptors with PGE_2_, although with different potencies.^10^, ^14^, ^15^ Based on these studies, we investigated whether PGE_3_ could engage this pathway to alternatively activate macrophages.

We explored the role of PGE_3_ in macrophage polarization and found that PGE_3_ selectively promoted M2a polarization, while inhibiting M1 and TAM polarization. Therefore, it exerted an inhibitory effect on prostate cancer inflammation. This process depended on PGE_3_‐induced up‐regulation of the PKA pathway.

## 2 MATERIALS AND METHODS

### 2.1 Cell culture

The human prostate cancer cell lines PC3 (ATCC, Cat. #: CRL1435), 22RV1 (ATCC, Cat. #: CRL2505;), and LNCaP (ATCC, Cat. #: CBP61040); human monocytic leukaemia cell line THP‐1 (ATCC, Cat. #: TIB‐202); and mouse fibroblast cell line L929 (ATCC, Cat. #: CCL‐1) were purchased from the Shanghai Institute of Cell Biology, Chinese Academy of Sciences (Shanghai, China). The PC3, 22RV1 and LNCaP cells were cultured in RPMI 1640 medium (Gibco) supplemented with 10% (v/v) foetal bovine serum (FBS, Gibco) at 37°C in an atmosphere of 5% CO_2_. THP‐1 cells were grown in RPMI 1640 medium supplemented with 10% FBS, 1% glutamine (Thermo Fisher Scientific) and 0.2 μmol/L β‐mercaptoethanol (Sigma‐Aldrich) at 37°C and 5% CO_2_.

### 2.2 Macrophage polarization

Polarization of THP‐1 cells into M0, M1, and M2 macrophages was performed as previously described.^16^ Briefly, THP‐1 cells were differentiated into M0 macrophages by incubation with 100 nmol/L phorbol12‐myristate 13‐acetate (PMA) for 24 hours (Sigma‐Aldrich). Once the cells were adherent, they were transferred to PMA‐free media for 24 hours (M0). These cells were then polarized to M1‐like macrophages by incubation with 100 ng/mL LPS (Sigma‐Aldrich) and 20 ng/mL IFN‐γ (R&D Systems) for 72 hours or M2a‐like macrophages by incubation with 20 ng/mL IL‐4 (R&D Systems) for 72 hours. At the same time, the cells were treated with or without PGE_3_ (100 nmol/L, Cayman Chemical).

Bone marrow‐derived macrophage (BED) isolation and cultivation were performed as described by the Cold Spring Harbor Protocols.^17^ Briefly, femur and tibia bones were collected from 6‐ to 8‐week‐old C57BL6/J mice, and then bone marrow cells were flushed out using phosphate‐buffered saline (PBS) supplemented with 2% heat‐inactivated FBS. After the red blood cells were lysed with red blood cell lysis buffer, the cells were cultured in BMDM growth medium for 7 days, followed by analysis of the purity of the cell population. BMDM growth medium contains 30% supernatant of L929 cells, which provides macrophage colony‐stimulating factor. To induce macrophage polarization, the cells were stimulated with LPS (100 ng/mL) and IFN‐γ (20 ng/mL) for M1 or IL‐4 (20 ng/mL) for M2a activation.

### 2.3 Co‐culture and differentiation of TAMs and conditioned medium preparation

To obtain M0 macrophages, THP‐1 cells were seeded at a density of 1 × 10^5^ cells per well in a six‐well culture plate and treated with 100 nmol/L PMA for 24 hours as previously described. Next, M0 cells in the six‐well plates were co‐cultured with PC3 cells that had been left to attach to the cell culture inserts for 12 hours before co‐culture. The cells were co‐cultured in RPMI 1640 supplemented with 10% FBS for 7 days and treated with or without PGE_3_ (100 nmol/L). The medium was replaced every 2 days. After 7 days, the medium was collected as the conditioned medium (CM‐control).

### 2.4 RNA extraction and quantitative real‐time PCR (qPCR)

Total RNA was extracted using TRIzol (Cat#15596026, Invitrogen) according to the manufacturer's protocol. Reverse transcription was performed according to the manufacturer's protocol with the Prime Script^®^ RT reagent Kit with gDNA Eraser (Cat#PR047A, Takara). Real‐time PCR was performed on a CFX96 Real‐Time System (Bio‐Rad Laboratories) using SYBR Green PCR master mix (Applied Biosystems) and the respective primer pairs for each gene (sequences presented in Table 1). All experiments were performed in triplicate, and data were normalized against beta‐actin. The relative mRNA expression of various genes is presented as the fold‐change, which was determined using the 2^‐ΔΔCT^ method as previously described.^18^

**TABLE 1:** Primers for quantitative real‐time PCR

Gene	Primer	Organisms
	Sequence (5’‐3’)	
Arg‐1	RE: TGGCTTGCGAGACGTAGAC FW: GCTCAGGTGAATCGGCCTTTT	Mouse
Fizz‐1	RE: GGTCCCAGTGCATATGGATGAGACC FW: CACCTCTTCACTCGAGGGACAGTTG	Mouse
IL‐10	RE: CGGTTAGCAGTATGTTGTCCAGC FW: CGGGAAGACAATAACTGCACCC	Mouse
iNOS	RE: CTGATGGCAGACTACAAAGACG FW: TGGCGGAGAGCATTTTTGAC	Mouse
TNFα	RE: GCTACGACGTGGGCTACAG FW: CCCTCACACTCAGATCATCTTCT	Mouse
IL‐6	RE: TTGGTCCTTAGCCACTCCTCC FW: TAGTCCTTCCTACCCCAATTTCC	Mouse
GAPDH	RE: TGTAGACCATGTAGTTGAGGTCA FW: AGGTCGGTGTGAACGGATTTG	Mouse
CD204	RE: TCCACTGAGAGGGATGAGAACT FW: TTCACTATCACAGGAGGACAC	Human
CD163	RE: AGCCATTATTACACACGTTCC FW: TTTTGTCACCAGTTCTCTTGGA	Human
TGFβ	RE: GAACCCGTTGATGTCCACTT FW: CACGTGGAGCTGTACCAGAA	Human
IL‐10	RE: GTGGGTGCAGCTGTTCTCAGACT FW: AAAAGAAGGCATGCACAGCTCAG	Human
CCL17	RE: CCCTGCACAGTTACAAAAACGA FW: GAGCCATTCCCCTTAGAAAGCT	Human
CD206	RE: CTACTGTTATGTCGCTGGCAAA FW: GGATGGAAGCAAAGTGGATTAG	Human
iNOS	RE: CACGGCCTTGCTCTTGTTTT FW: GTGATGCCCCAAGCTGAGA	Human
TNFα	RE: GGCCAGAGGGCTGATTAGAGA FW: CTTCTGCCTGCTGCACTTTG	Human
CXCL3	RE: GTGGCTATGACTTCGGTTTGG FW: TGCCAGTGCTTGCAGACACT	Human
IL‐6	RE: TCTGAGGTGCCCATGCTACATTT FW: GCTGCAGGACATGACAACTCATC	Human
CXCL9	RE: GTCCCTTGGTTGGTGCT FW: CATCTTGCTGGTTCTGATTGGA	Human
CCR7	RE: GTAATCGTCCGTGACCTCATCTT FW: GCTGGTGGTGGCTCTCCTT	Human
GAPDH	RE: GCCAGTAGAGGCAGGGATGATGTTC FW: CCATGTTCGTCATGGGTGTGAACCA	Human
EP1	RE: ACAGGCCGAAGAAGACCAT FW: CCATGTTCGTCATGGGTGTGAACCA	Human
EP2	RE: CGAGACGCGGCGCTAATAGA FW: CGAGACGCGACAGTGGCTTCC	Human
EP3	RE: ATGGCTCTGGCGATGAACAACGAG FW: CGGGGCTACGGAGGGGATGC	Human
EP4	RE: AGCCCTATCGGAAGGGTTGA FW: CCTTCGACGCACAATGCTTG	Human

### 2.5 Flow cytometry

Cells were detached using 0.05% trypsin (Gibco) and re‐suspended in PBS. Cell suspensions were then stained with anti‐CD206 or CCR7 monoclonal antibodies for 30 minutes at 4°C after FcγRII/III blocking. Flow staining buffers and antibodies were purchased from different companies, with detailed information listed in Table 2. Isotype‐matched controls were included in all experiments. Stained cells were analysed on an Attune NxT flow cytometer (Thermo Fisher Scientific).

**TABLE 2:** Antibodies for flow cytometry analysis

Antibody	Company	Catalog	Organisms
APC anti‐human CD206	BioLegend	321110	Human
PE iNOS Antibody (4E5)	Novus	NBP2‐22119	Human
PE anti‐mouse CD206	BioLegend	141705	Mouse
APC ANTI‐MOUSE NOS2 (CXNFT)	eBioscience	17‐5920‐80	Mouse
FITC ANTI‐MOUSE CD11B (M1/70)	eBioscience	11‐0112‐81	Mouse

### 2.6 Tumour transplantation in nude mice

Nude mice (BABL/c) were inoculated with a mixture of cells and Matrigel (BD Matrigel^™^ Basement Membrane Matrix, Cat#354248, BD Biosciences) on both sides of the back at 5 weeks of age. All procedures were approved by the ethics committee of Jiangnan University (protocol number: JN.NO20191030b048130[289]). The PC3 xenograft model was selected. PC3 cells were cultured to a density of 80% (logarithmic growth phase), and cell passage experiments were performed. Nude mice were transplanted with 1 × 10^7^ cells/mL in serum‐free RPMI 1640, and then mixed with an equal volume of Matrigel, which had been thawed on ice overnight. Vaccination was performed as soon as possible after mixing (30 minutes). Each nude mouse was inoculated with 50 μL of cell and Matrigel mixture subcutaneously on the back on each of the left and right sides, and tumour growth was regularly observed thereafter. Successfully transplanted nude mice (uniform tumour size) were divided into control and PGE_3_ groups with 6 mice in each group. In the PGE_3_ group, 1 μmol/L PGE_3_ (diluted in 50 μL saline) was injected next to the tumour, whereas the control group was injected with the same volume of saline near the tumour at the same time point. All treatments were performed every 3 days. Four weeks later, nude mice were sacrificed by cervical dislocation. Tumour tissues were removed and weighed, and then embedded into optimal cutting temperature (OCT) compound, cut into 8‐µm‐thick sections. Immunofluorescent staining was performed with rabbit anti‐CD68 (Cat. #: 2808‐1‐AP, Proteintech) and mouse anti‐CD206 (Cat. #: 60143‐1‐Ig, Proteintech) antibodies. All primary antibodies were incubated with the sections at 4°C overnight, followed by anti‐rabbit IgG (Cat. #: ab150077, Abcam) and anti‐mouse IgG (Cat. #: SA00006‐3, Proteintech) secondary antibodies. All sections were then incubated with DAPI. Images were captured using an inverted fluorescence microscope (Nikon ECLIPSE Ti‐S), and the total number and positively stained cells were calculated with ImageJ software (NIH).

### 2.7 Acute inflammation model

All procedures were approved by the ethics committee of Jiangnan University (protocol number: JN. No20191115c1081230[306]). After 1 week of adaptation, 6‐ to 8‐week‐old C57BL6/J mice were divided into 3 different groups (6 in each group) and injected intraperitoneally with saline, LPS (5 mg/kg), or LPS + PGE_3_ (5 mg/kg + 1 μmol/L). After 24 hours, blood was collected retroorbitally and the mice were sacrificed. Mouse peritoneal macrophages (MPMs) were obtained from peritoneal lavages with PBS for further analysis.

### 2.8 Enzyme‐linked immunosorbent assay (ELISA)

Sandwich ELISAs were performed to detect cytokines in the serum and MPMs of LPS‐stimulated mice. ELISA kits for IL‐6 and IFN‐ γ were obtained from R&D Systems. The serum was directly used for ELISAs in 96‐well plates without dilution. MRMs were diluted to 1 × 10^7^ cells/mL in PBS, sonicated, and centrifuged; the supernatant was used for ELISA. All procedures were performed according to the manufacturer's protocol. Cytokine concentrations were calculated using to standard curves.

### 2.9 Phagocytosis assay

The phagocytosis is detected by Flow cytometry. PC3 cells were detached using 0.05% trypsin (Gibco) and washed with PBS three times. Cells were re‐suspended to 1 × 10^7^ cells/mL in PBS, stained with CFSE (Cat. #: HY‐D0938, Med Chem Express). Supernatant of TAM was replaced with CFSE‐stained PC3 cell suspension, and the co‐culture was incubated for 30 minutes. After discarding supernatant, cells were detached, washed three times with PBS, stained with anti‐CD206 antibody. CFSE and CD206 double‐positive cell population was analysed by Flow cytometry.

The phagocytosis is detected by Neutral red solution. After discarding supernatant, TAM cells (in 96‐well plate) would be washed with PBS three times. The cells were then incubated with 0.1% neutral red solution (Cat. #: R22255, Shanghai yuanye Bio‐Technology) for 20 minutes, after which the media were removed carefully and the cells were washed with PBS five times. Then, the cells were solubilized in 0.2 mL solution (50% ethanol, 49% water and 1% acetic acid). Optical absorbance was measured at 540 nm 4 hours later.

### 2.10 Proliferation assay

3‐(4,5‐Dimethylthiazol‐2‐yl)‐2,5‐diphenyltetrazolium bromide (M2128, Sigma‐Aldrich) was prepared by dissolving 5 mg M2128 in 1 mL PBS. The stock solution was protected from light and stored at −20°C. To evaluate proliferation, PC3 cells (1.5 × 10^3^ cells) were seeded into 96‐well plates in sextuplicate and allowed to adhere overnight in complete RPMI 1640. The medium was then removed and replaced by FBS‐free medium for 24 hours, after which the cells were cultured in conventional medium, CM‐control with or without PGE_3_, or CM/PGE_3_ for 72 hours. At various time points, the medium was removed and replaced by 100 µL FBS‐free medium with 10 µL of prepared MTT solution. The cells were then incubated at 37°C for 4 hours, after which the media were removed carefully and the cells were solubilized in 0.2 mL dimethyl sulphoxide. Optical absorbance was measured at 570 nm using a reference wavelength of 630 nm. Data were calculated as the OD570‐OD630, and cell numbers were reported as percentages compared to the control.

### 2.11 Western blotting

Protein samples were separated by 10% SDS‐PAGE and then transferred to polyvinylidene difluoride membranes (MA01821, Millipore). The membranes were blocked for 2 hours in 5% skim milk and then incubated with primary antibodies (Table 3) overnight at 4°C. Horseradish peroxidase‐labelled goat anti‐mouse or anti‐rabbit IgG secondary antibodies were used for ECL detection (WBKLS0500, Sigma‐Aldrich).

**TABLE 3:** Antibodies for Western blot

Antibody	Company	Catalog	Organisms
Anti‐h/m/r PKA Cα/β	R&D Systems	MAB5908	Human
Phospho‐PKA C (Thr197) Antibody	Cell Signaling Technology	4781S	Human
PKC Beta Antibody	Proteintech	12919‐1‐AP	Human
Phospho‐PKC (pan) (βII Ser660) Antibody	Cell Signaling Technology	9371S	Human
Akt Antibody	Cell Signaling Technology	9272	Human
Phospho‐Akt (Ser473) Antibody	Cell Signaling Technology	9271	Human
β‐tubulin	BosterBio	BM1453	Human
β‐actin	Sigma	A1978	Human
Anti‐COX2	Abcam	ab1591	Human

### 2.12 Statistics

All data were analysed using GraphPad Prism 6 software. All data are shown as the means ± SD. One‐way analysis of variance was performed to determine the significance among three or more groups followed by the indicated post hoc tests. *t* test was used to analyse two independent samples. *P* < .05 was considered as statistically significant.

## 3 RESULTS

### 3.1 Prostaglandin E_3_ suppresses M1 markers and induces M2a marker expression in polarized macrophages

**FIGURE 1:**
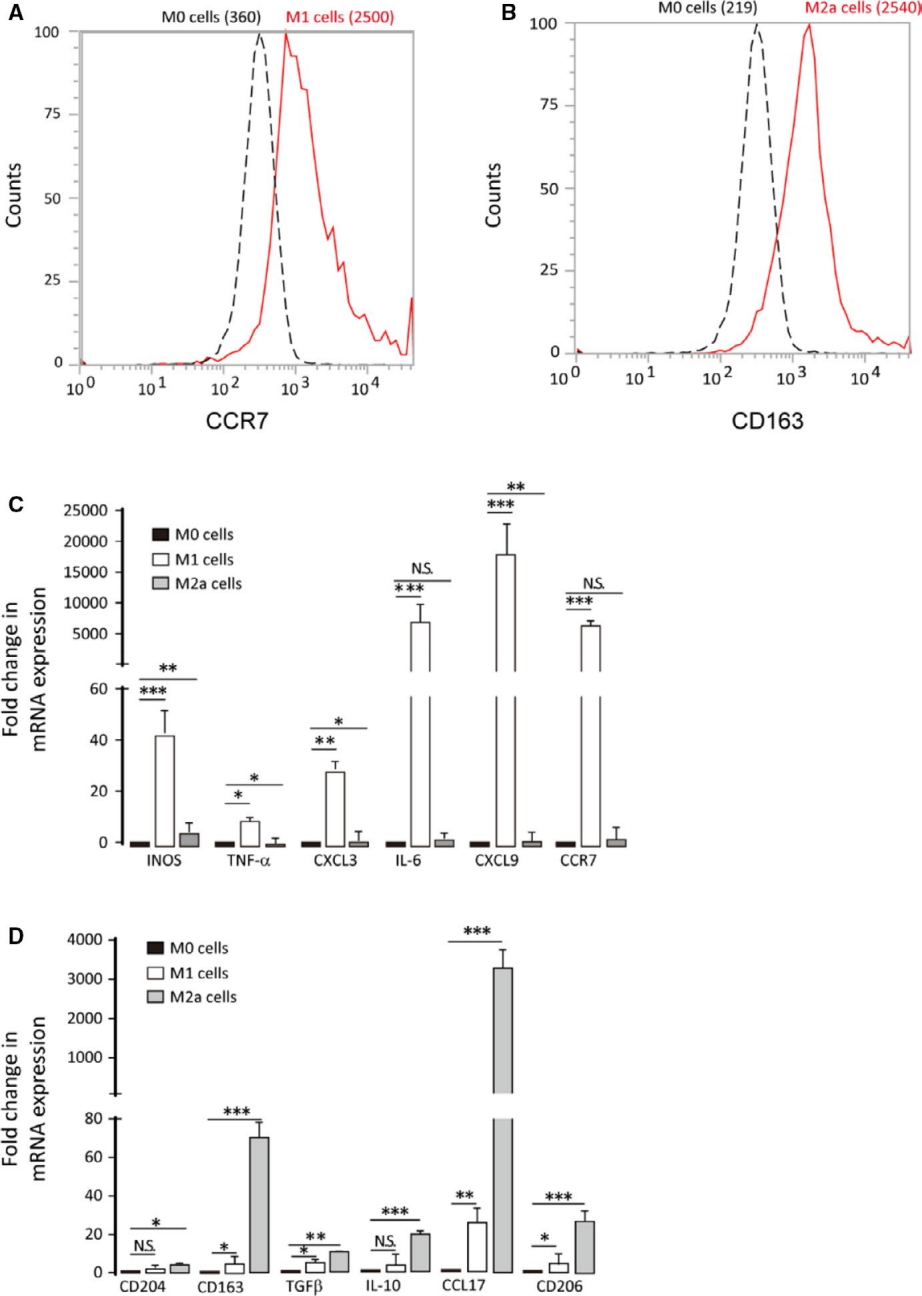
THP1 cells can be polarized to M1/M2a macrophages. M0 cells were obtained by treating THP‐1 cells with PMA for 24 h, followed by incubation in conventional media for 24 h. M0 cells were polarized to M1 or M2a cells using 100 ng/mL LPS and 20 ng/mL IFN‐γ or with 20 ng/mL IL‐4 for 72 h, respectively. A‐B, M1 marker (CCR7) and M2a marker (CD163) expression were analysed by flow cytometry. C‐D, qPCR analysis of M1 (iNOS, TNFα, CXCL3, IL6, CXCL9 and CCR7) and M2a (CD204, CD163, TGFβ, IL10, CCL17 and CD206) markers. Graphs represent means ± SD; *P ≤ .05, **P ≤ .01, ***P ≤ .001

Prostaglandins are potent lipid molecules affecting vital aspects of immunity.^19^ We predicted that PGE_3_ affects the immune microenvironment. We used THP‐1 cells as an in vitro macrophage model.^16^ qPCR and flow cytometry were performed to measure the expression of classical M1 and M2a markers. Following polarization, we observed marked up‐regulation of CCR7 in M1 cells and CD163 in M2a cells compared to in M0 cells (Figure 1). Consistent with these findings, iNOS, TNF‐α, CXCL3, IL6, CXCL9 and CCR7 showed significantly higher expression in M1 cells, and CD204, CD163, TGF‐β, IL10, CCL17 and CD206 showed significantly higher expression in M2a cells compared to in M0 cells (Figure 1).

**FIGURE 2:**
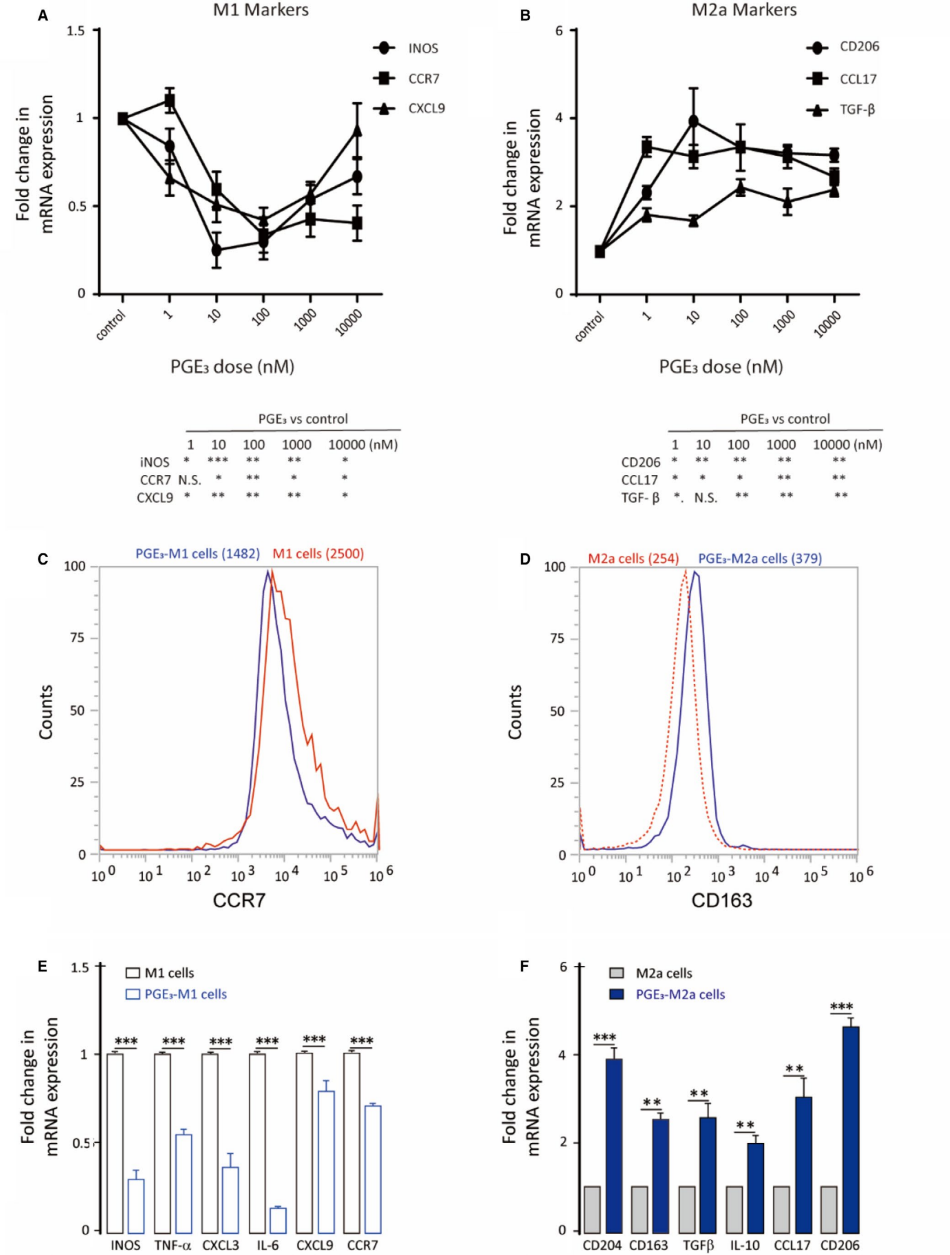
Prostaglandin E_3_ suppresses M1 and induces M2a marker expression in polarized macrophages. A‐B, Dose–response of M1 and M2a markers after PGE3 treatment (1‐10 000 nmol/L). C, CCR7 protein expression in THP1‐derived M1 macrophages following PGE_3_ treatment (100 nmol/L). D, CD163 protein expression in THP1‐derived M2a macrophages following PGE_3_ treatment. E‐F, M1 and M2a marker expression following PGE_3_ treatment. Graphs represent means ± SD; NS, No significance, *P ≤ .05, **P ≤ .01, ***P ≤ .001

**FIGURE 3:**
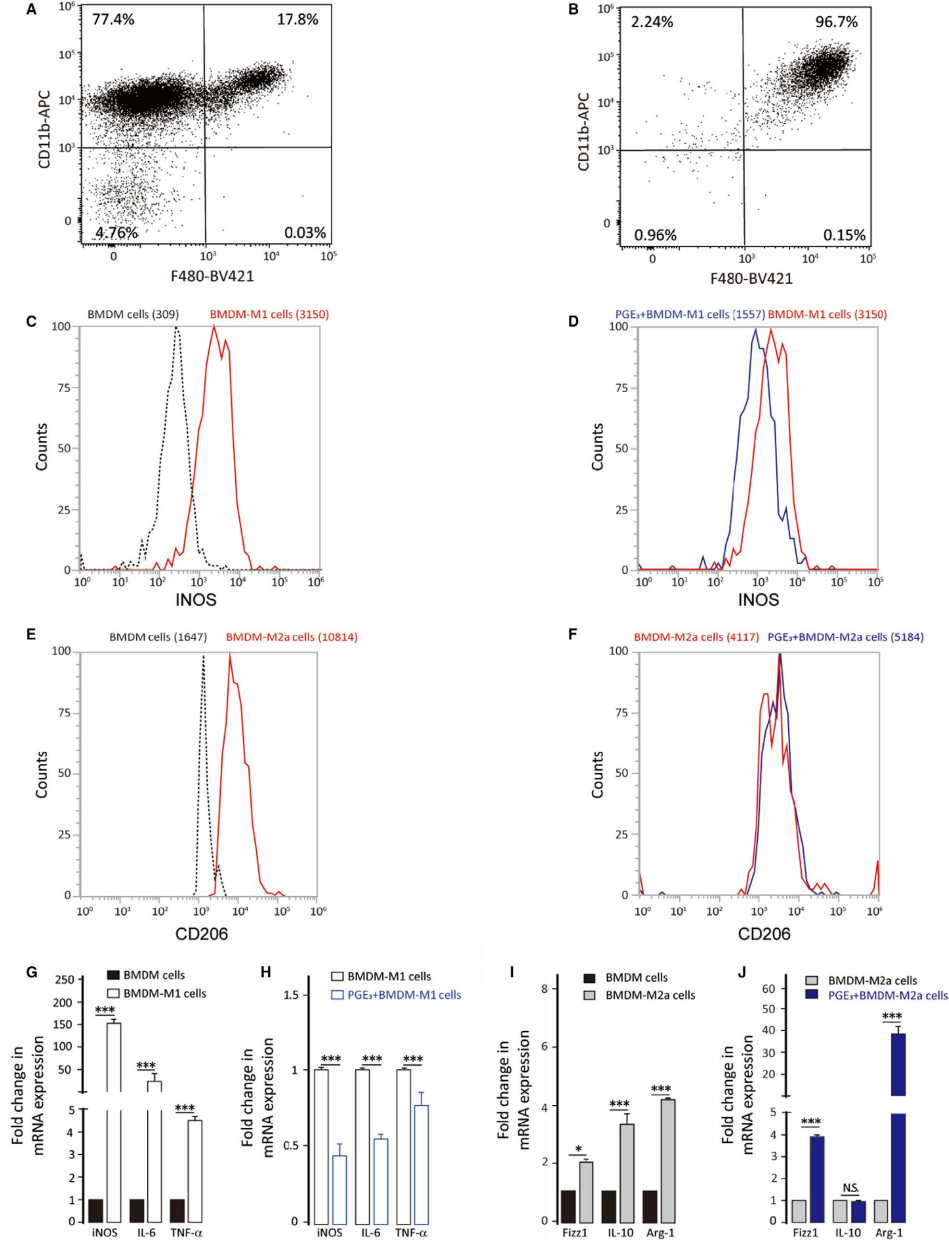
Effect of PGE_3_ treatment on bone marrow‐derived macrophage polarization. BMDM cells were treated with 100 ng/mL LPS plus 20 ng/mL IFN‐γ or treated with 20 ng/mL IL‐4 for 24 h in the presence or absence of PGE_3_. A, BMDM cells were cultured in RPMI 1640 medium. B, BMDM cells were cultured in RPMI 1640 medium with 30% L929 cell supernatant. C‐F, M1 marker (iNOS) and M2a marker (CD206) expression were analysed by flow cytometry. G‐H, qPCR analysis of M1 (iNOS, IL6, TNFα) and M2 (Fizz1, Arg‐1, IL10) markers. Graphs represent means ± SD; NS, No significance, *P ≤ .05, ***P ≤ .001

To evaluate potential functions of PGE_3_ in macrophages, M0 cells were treated with PGE_3_ (100 nmol/L) during polarization. PGE_3_ caused down‐regulation of M1 markers and up‐regulation of M2a markers (Figure 2). To confirm these findings, BMDMs were isolated and collected from C57BL/6 mice, as previously described.^20^ Most BMDMs (96.7%) were macrophages after culture with 30% L929 cell supernatant for 7 days under our experimental conditions (Figure 3A, B). BMDMs were polarized to M1 or M2a phenotypes by LPS and IFN‐γ or IL‐4 treatment, respectively. Similar to the results in THP‐1 cells, PGE_3_ up‐regulated M2a markers and down‐regulated M1 markers (Figure 3C‐J). These results suggest that PGE_3_ preferentially inhibits polarization towards M1 but promotes polarization of M2a macrophages.

### 3.2 PGE_3_ has anti‐inflammatory effects in vivo

**FIGURE 4:**
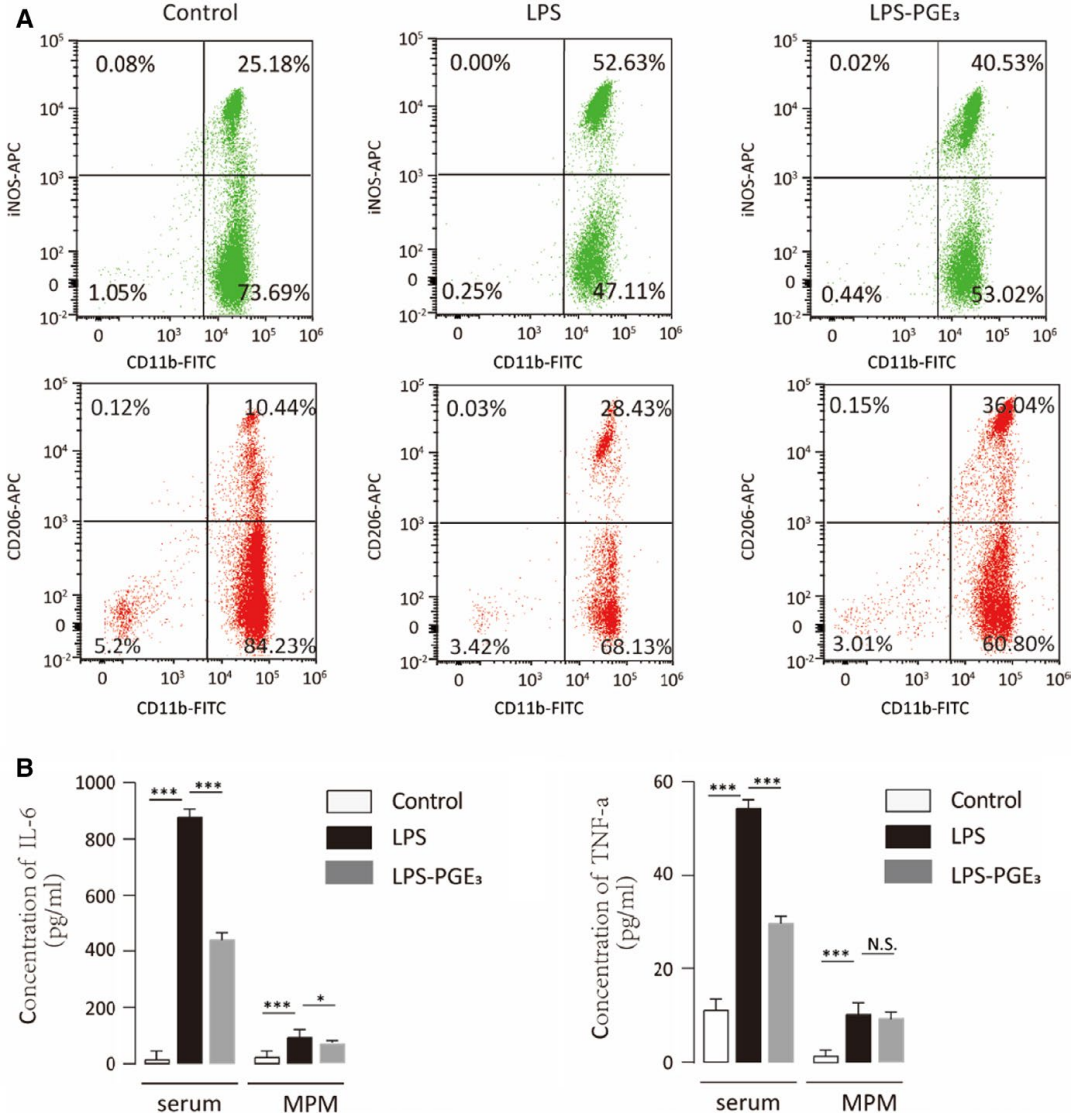
Prostaglandin E_3_ inhibits inflammation in vivo. C57BL6/J mice were stimulated with LPS to induce acute inflammation. Mice in the control group were injected with saline and those in the LPS‐PGE_3_ group were injected with LPS and PGE_3_ together. A, After 24 h, mouse peritoneal macrophages (MPMs) were isolated and analysed by flow cytometry. B, ELISA was performed to determine the concentrations of IL‐6 and TNF‐α in both serum and MPM lysates. Graphs represent means ± SD; NS, No significance, *P ≤ .05, ***P ≤ .001

We next evaluated the role of PGE_3_ during acute inflammation in vivo. Mice 6‐8 weeks old were divided into 3 different groups (6 in each group) and injected intraperitoneally with saline, LPS or LPS + PGE_3_. After 24 hours, PGE_3_ significantly reduced MPMs with an M1‐like phenotype and increased M2‐like MPMs (Figure 4A). Additionally, PGE_3_ inhibited inflammatory cytokine secretion in both the serum and MPM cell lysates (Figure 4B). These results indicate that PGE_3_ can significantly (*P* < .05) modulate macrophage polarization towards an anti‐inflammatory function.

### 3.3 Prostaglandin E_3_ suppresses TAM polarization and enhances the phagocytosis of TAM

**FIGURE 5:**
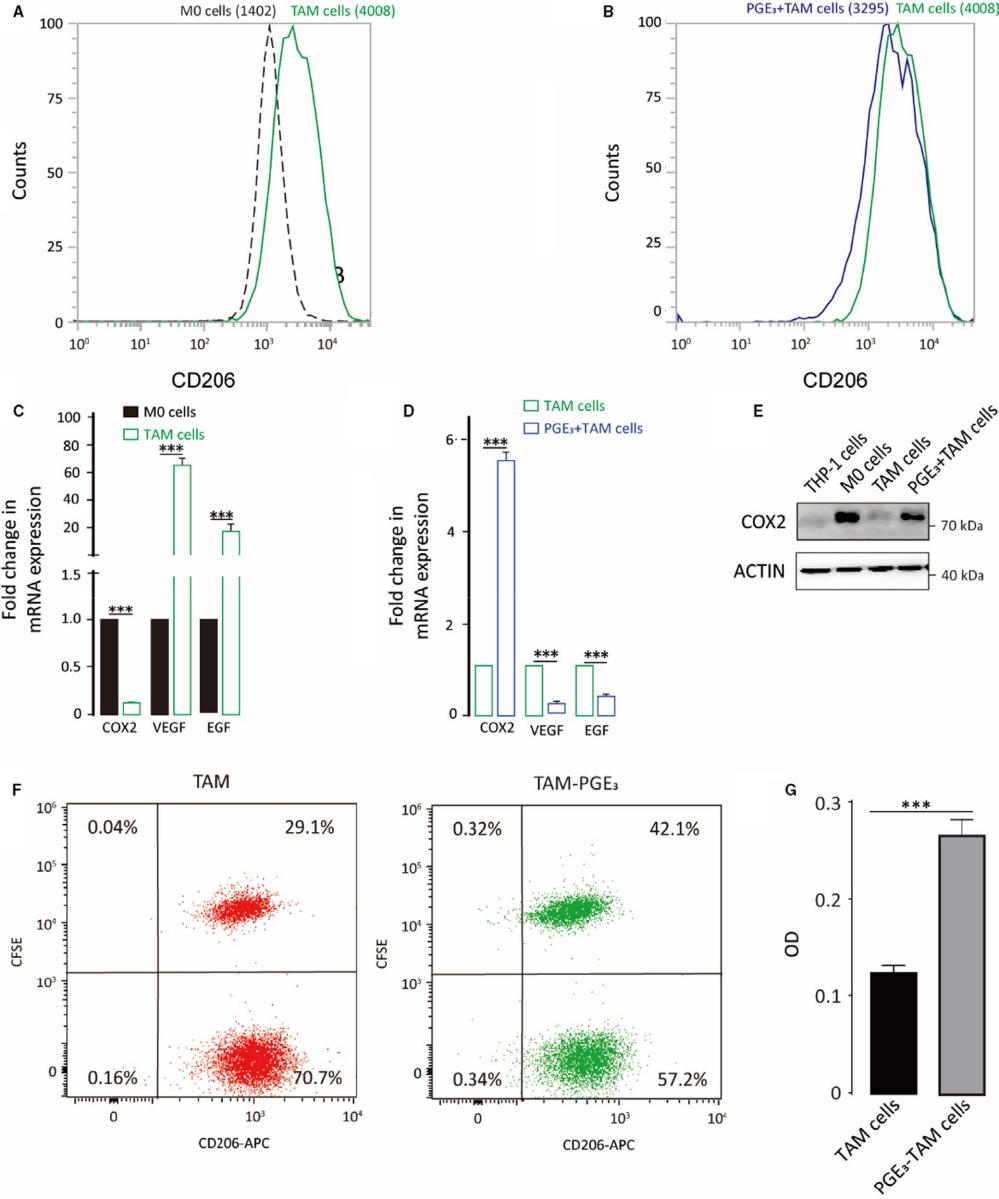
Prostaglandin E_3_ suppresses TAM polarization and enhances the phagocytosis of TAM. TAMs were derived from THP‐1 cells co‐cultured with PC3 cells. A, CD206 protein expression on macrophages was analysed by flow cytometry. B, CD206 expression in TAMs following PGE_3_ treatment (100 nmol/L). C‐D, TAM cell markers were detected by qPCR compared to M0 cells, and TAM markers were measured following PGE_3_ treatment. E, COX2 protein expression following PGE_3_ treatment. F, Phagocytosis of TAM detected by Flow cytometry. G, Phagocytosis of TAM detected by Neutral red solution. Graphs represent means ± SD; ***P ≤ .001

TAMs (also known as M2d) reside in the tumour microenvironment promote tumour cell migration and invasion; these cells are a unique subtype of M2 macrophages.^21^ Although PGE_3_ promoted M2a polarization, the role of PGE_3_ in TAM polarization is unclear. TAMs were induced by co‐culturing THP‐1 cells with PC3 prostate cancer cells. The canonical M2 macrophage marker CD206 was up‐regulated in THP‐1‐derived TAMs (Figure 5A). qPCR also revealed down‐regulation of COX2 and up‐regulation of VEGF and EGF in THP‐1 TAM cells compared to in M0 cells (Figure 5C). In contrast, PGE_3_ promoted the expression of COX2 and down‐regulated the expression of CD206, VEGF and EGF (Figure 5B, D, and E), suggesting that PGE_3_ suppresses TAM polarization.

As inducing phagocytosis of macrophages is a therapeutic strategy in clinic,^22^ we also performed the phagocytosis assay to better corroborate the effect of PGE_3_ on TAM. Results showed that PGE_3_ treatment enhanced the phagocytosis (Figure 5F and G), suggesting that PGE_3_ might reduce tumour growth by increasing TAM phagocytosis.

### 3.4 Prostaglandin E_3_ suppresses prostate tumour cell proliferation

**FIGURE 6:**
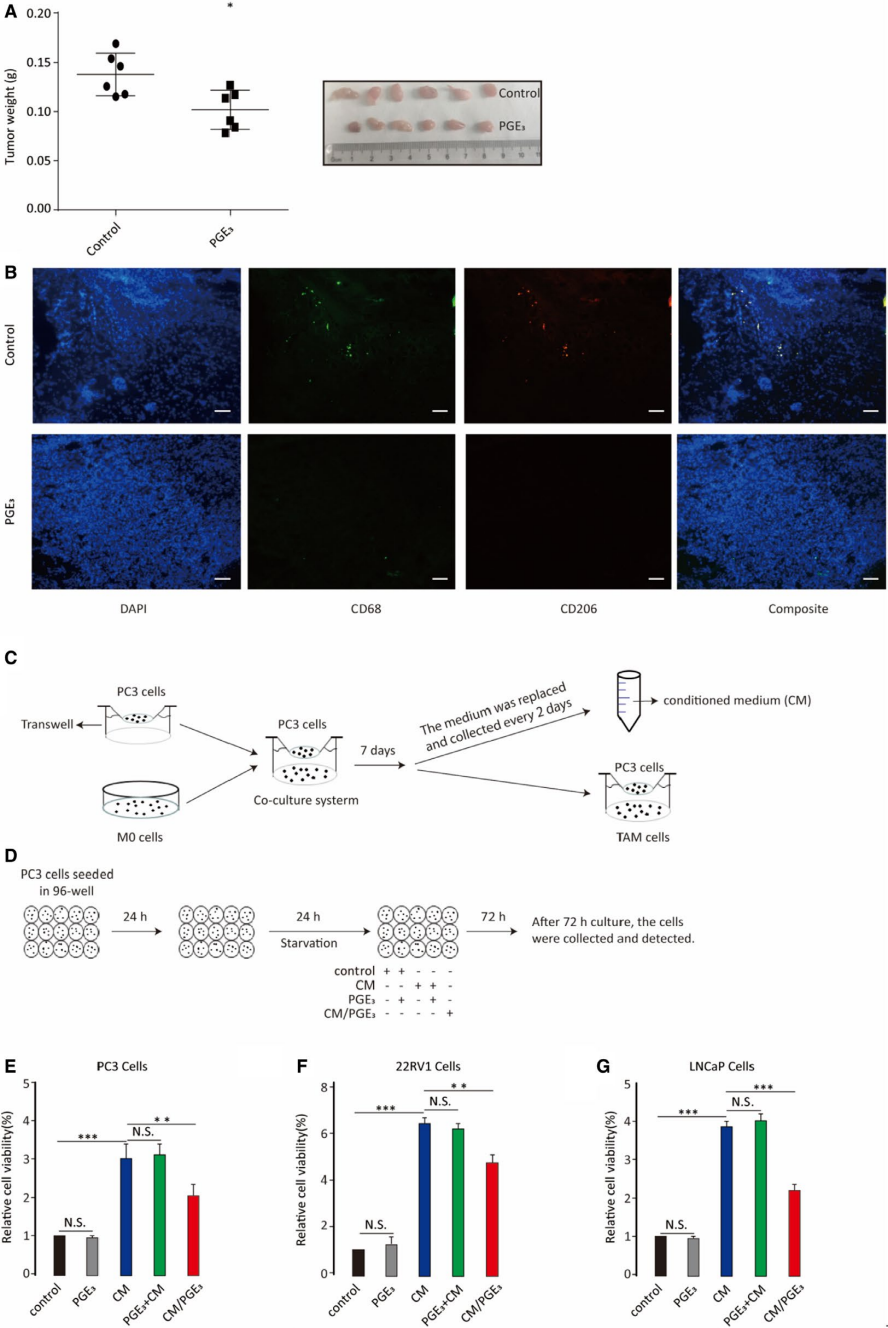
Prostaglandin E_3_ suppresses prostate tumour growth by down‐regulating TAM polarization. A, Transplanted tumour weights with or without PGE_3_ treatment. B, Measurement of TAM infiltration by immunofluorescence staining of CD68 and CD206. C, Flow chart of the co‐culture system, differentiation of TAMs, and preparation of conditioned media (CM). PC3 cells were co‐cultured with M0 cells for 7 d to differentiate into TAMs. The co‐culture medium was replaced every 2 d and collected as the CM. During the co‐culture, one group had PGE_3_ added, with its collected CM defined as CM/PGE_3_. D, For the proliferation assay, PC3 cells were seeded onto 96‐well plates for 24 h to adhere before stimulation. Media were then removed from PC3 cells and replaced by conventional medium, CM‐control with or without PGE_3_, and CM‐PGE_3_, after serum starvation as described previously. After 72 h, the cells were collected and analysed. E‐J, Analysis of proliferation of prostate tumour cells (PC3, 22RV1 and LNCaP cells) treated with CM, CM/PGE_3_, CM with PGE_3_ added (PGE_3_+CM) or PGE_3_ alone compared to the conventional medium (control). Graphs represent means ± SD; NS, No significance, **P ≤ .01, ***P ≤ .001

To investigate the role of PGE_3_ in tumorigenesis, we performed tumour transplantation experiments and found that PGE_3_ decreased the weights of transplanted tumours after 1 month of continuous PGE_3_ injection (Figure 6A). As TAMs always show M2‐like phenotypes, we measured CD68 (a total macrophage marker) and CD206 (an M2‐like macrophage marker) expression in the tumours and observed that both CD68 and CD206 expression can be inhibited by PGE_3_ (Figure 6B **)**. These results suggest that PGE_3_ has antitumorigenic effects by regulating macrophage polarization in vivo. Next, we tested whether PGE_3_ also inhibits the proliferation of cancer cells in vitro in a co‐culture system with THP‐1 cells. CM with or without PGE_3_ (CM/PGE_3_) was collected (Figure 6C) and used in cell proliferation assays (Figure 6D).

We evaluated three prostate cancer cell lines (PC3, 22RV1, and LNCaP) to test our hypothesis. CM significantly promoted the proliferation of prostate tumour cells compared to control, whereas CM/PGE_3_ was less able to stimulate proliferation. Importantly, addition of PGE_3_ either into CM or directly onto tumour cells did not inhibit tumour cell proliferation (Figure 6E‐J). These results suggest that PGE_3_ suppresses tumour cell proliferation by inhibiting TAM polarization. We also analysed the cell cycle in PC3 cells under the above conditions and found that PGE_3_ reversed the proliferation of TAM on PC3 cells (Figure S1).

### 3.5 Prostaglandin E_3_ influences macrophage polarization through the PKA pathway

**FIGURE 7:**
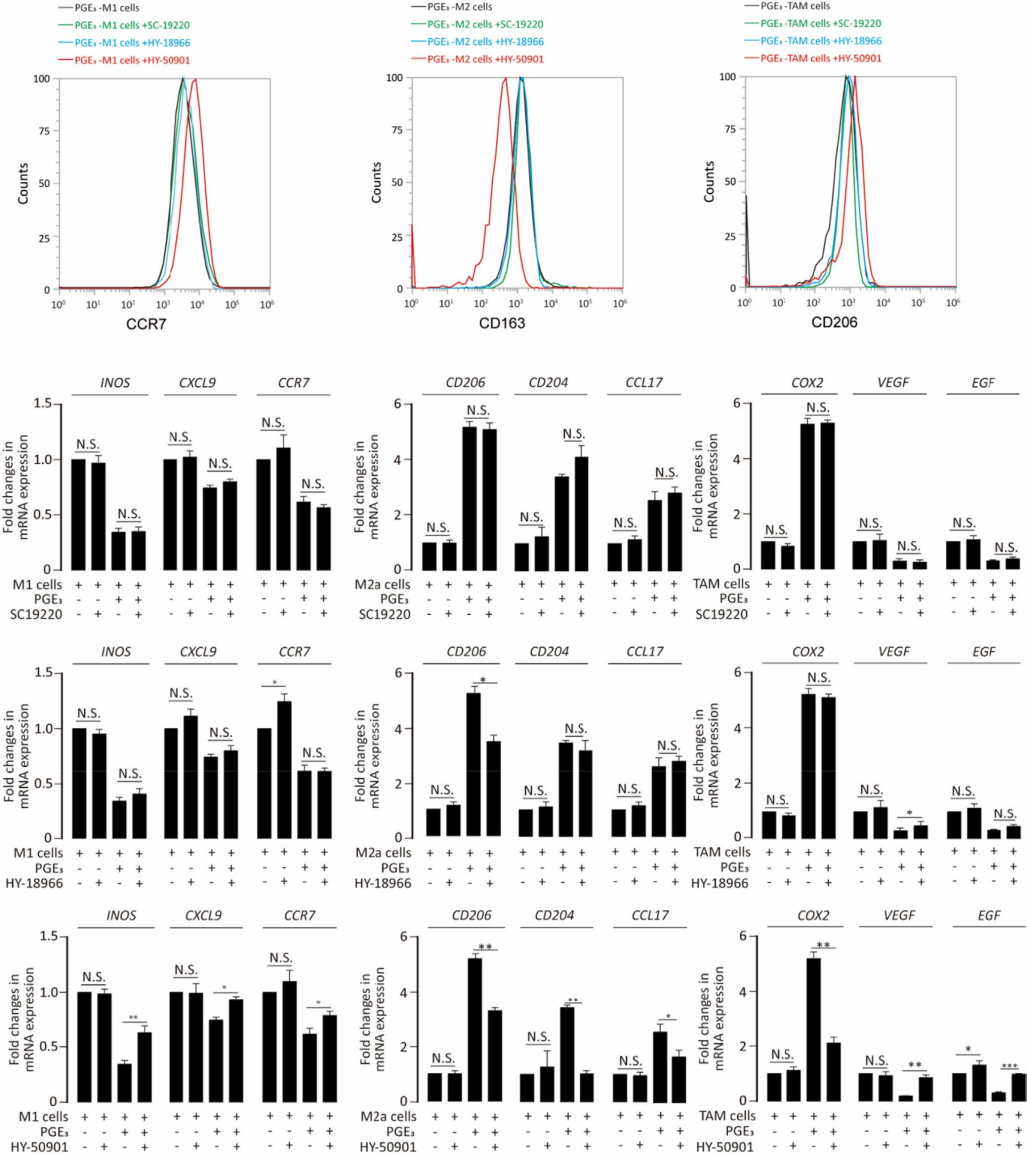
Prostaglandin E_3_ modulates macrophage polarization via EP receptor. Different EP receptor antagonists were added to examine which was (were) involved in the polarization of macrophages. SC19220 was the antagonist of EP1 receptor. HY‐18966 was the antagonist of EP2 receptor. HY‐50901 was the antagonist of EP4 receptor. Graphs represent means ± SD; NS, No significance, *P ≤ .05, **P ≤ .01

Normally, prostaglandins affect target cells by activating their cognate receptors EP1, EP2, EP3 or EP4. Expression of EP1, EP2 and EP4 was detectable by RT‐PCR in THP‐1 cells **(**Figure S2A). EP1 is coupled to G_q/p_ and induces PKC activation by mobilizing intracellular calcium. EP2 and EP4, however, are coupled with G_s_ and induce PKA by up‐regulating cAMP.^19^ Previous studies showed that EP4 can activate protein kinase B, also known as AKT.^23^ We detected the expression of PKA, PKC and AKT and their phosphorylation states by Western blotting. The results are shown in Figure S2B. To determine which EP subtype(s) was (were) involved in the polarization of macrophages, we did a series of experiments using receptor antagonists. Results showed that EP4 mediated the PGE_3_‐induced macrophage polarization (Figure 7 and Figure S3).

**FIGURE 8:**
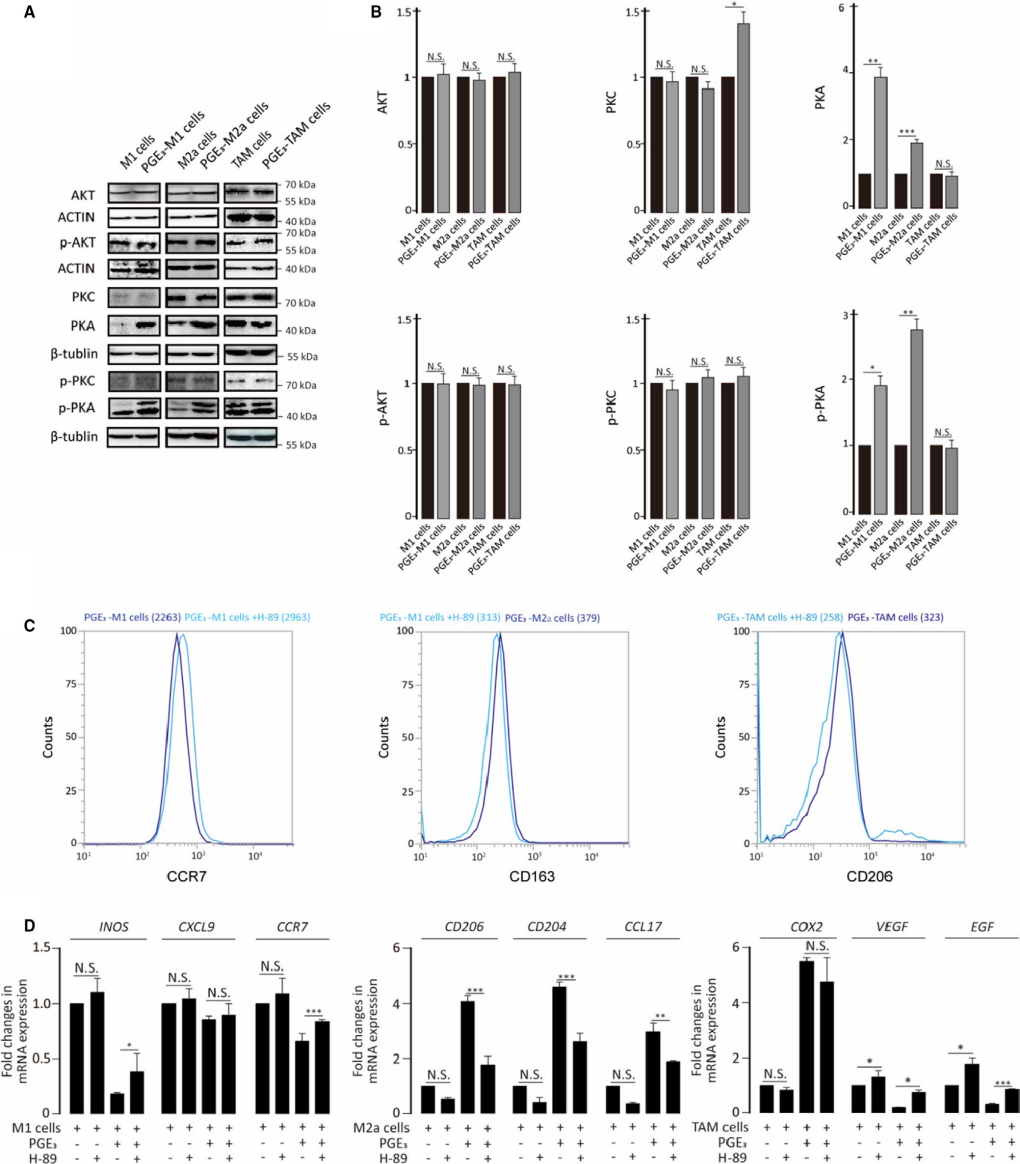
Prostaglandin E_3_ regulates the expression of PKA. A‐B, Expression of AKT, PKC, PKA and their phosphorylation states in M1, M2a or TAM cells in the presence or absence of PGE_3_. C, Measurement of M1 (CCR7) or M2a (CD163, CD206) markers after treatment with H‐89 (500 nmol/L) D, Analysis of M1 and M2a markers by qPCR after treatment with H‐89 treatment

Further analyses showed that both AKT and p‐AKT (Ser473 phosphorylation) did not differ in M0, M2a and TAMs, even when stimulated with PGE_3_ (Figure 8A, B). PKC‐β and p‐PKC (βII Ser660 phosphorylation) showed low expression in M1 cells, and PGE_3_ was unable to change their expression; a similar trend was observed in M2a cells. In contrast, PKC levels were slightly increased when TAMs were treated with PGE_3_, although PKC phosphorylation showed no changes. Interestingly, the expression of both PKA and p‐PKA (Thr197 phosphorylation) was up‐regulated when M1 or M2a cells were treated with PGE_3_. However, these results were not affected by PGE_3_ treatment of TAMs (Figure 8A, B).

As our results showed that PGE_3_ up‐regulated the expression of PKA in both M1 and M2a cells, we predicted that PGE_3_ influences macrophage polarization via PKA. To test this mechanism, we added a potent and selective PKA inhibitor (H‐89 dihydrochloride) during macrophage polarization and found that the immunomodulatory effects of PGE_3_ on macrophages were inhibited by H‐89 dihydrochloride (Figure 8C, D).

## 4 DISCUSSION

Inflammation is considered as a significant contributor to many human diseases, including cancer. Macrophages are often considered as key components of sites of inflammation and tumour microenvironments. The functions of macrophages are closely related to their polarization. M1‐like macrophages contribute to inflammation ^24^, ^25^ by releasing high levels of pro‐inflammatory cytokines, including TNF‐α and IL‐6.^26^ In contrast, M2‐like macrophages can prevent inflammation ^27^, ^28^ and express high levels of mannose receptor (CD206), CD163,^29^ IL‐10 and arginase‐1.^30^

Prostaglandins are endogenous substance with extensive biological activities, which are derived from C_20_ polyunsaturated fatty acids. According to the number of double bonds in the fatty acid side chains, the prostaglandins are divided into 1, 2 and 3 series prostaglandins. Prostaglandin‐1, −2 and −3 are derived from dihomo‐γ‐linolenic acid (DHLA), arachidonic acid (AA) and eicosapentaenoic acid (EPA), respectively.^31^, ^32^ It is reported that the 3‐series prostaglandins tend to have anti‐inflammatory and anti‐tumour effects.^10^, ^33^, ^34^ In our research, we found that Prostaglandin E_3,_ as a classic 3‐series prostaglandins, can regulate the polarization of M1 and M2a macrophages by inhibiting M1 and promoting M2a macrophage polarization, supporting its proposed anti‐inflammatory effects.

Macrophages are a major component of tumour‐infiltrating inflammatory cells,^21^, ^35^ and TAMs contribute to tumour progression at different levels.^36^ Previous studies reported that some anti‐inflammatory and anti‐cancer effects of EPA are mediated by PGE_3_ production,^13^, ^37^ but the mechanisms are unclear. Our study revealed that PGE_3_ acts on macrophages directly rather than on tumour cells in the co‐culture system. This result was confirmed in vivo. These findings suggest that the anti‐tumorigenic effects of EPA are at least partly because of its conversion to PGE_3_, and that PGE_3_ exerts its anti‐tumorigenic effects during many stages of tumour development.

Studies have shown that newly formed PGs exert their functions by binding to their receptors ^38^; however, although the receptor for PGE_2_ is well‐defined, information on the receptor for PGE_3_ remains limited. Some evidence suggests that PGE_3_ shares the same EP receptor system with PGE2 but in these molecules, they have different binding affinities and potencies.^10^ The cognate receptors EP1, EP2, EP3 and EP4^11^, ^33^ are coupled to different G proteins and use different second messenger signalling pathways.^39^ EP1 is coupled to G_q/p_ and induces activation of protein kinase C through by mobilizing intracellular calcium, whereas EP2 and EP4 are coupled with G_s_ proteins and induce the expression of cAMP, leading to PKA regulation. EP3, however, is typically coupled to G_i_ proteins and inhibits cAMP. Our study showed that all EP receptors, except for EP3, were expressed in THP‐1 cells. Moreover, we founded that only the antagonist of EP4 receptor could stop the polarization by PGE3. We also found that both phosphorylated and total PKA in M1 and M2a macrophages were increased by PGE_3_. These results suggest that PGE_3_ activates a signalling pathway through the EP4 receptor.

Macrophage polarization and functions are tightly regulated by several interconnected pathways.^40^ Among these, activation of STAT1 and STAT3/STAT6 has been demonstrated to play a crucial role,^41^, ^42^, ^43^ with the downstream effector KLF‐4 promoting M2 macrophage functions.^44^ Previous studies have linked the CREB/Kruppel‐like factor 4 pathway to macrophage polarization and reported that PGE_2_ can promote M2 polarization through a cAMP/PKA/CREB‐dependent pathway.^12^, ^45^ Thus, PGE_3_ may influence macrophage polarization via an EP/PKA‐dependent pathway to achieve anti‐inflammatory and anti‐tumorigenic effects. However, the mechanisms underlying TAM polarization remain unclear. Although we did not identify the factor(s) initiating TAM polarization, there was a slight increase in PKA levels in TAM; therefore, additional studies are needed to explore how PKA activation contributes to this polarization. We also found that PGE_3_, as well as the PKA inhibitor H‐89, can partially blunt TAM polarization. These results suggest that PKA activation influences TAM polarization, but the effect of PGE_3_ may be multi‐targeted. As the co‐culture system is complex, multiple factors likely influence the differentiation of TAMs, and identifying these factors will be the focus of our future experiments.

Given that prostaglandins are natural ligands of peroxisome proliferator‐activated receptor (PPAR) γ, which plays a crucial role in the development of inflammation^46^, ^47^ and differentiation of macrophage,^48^ we determined the effect of PPAR‐**γ** activation on macrophage polarization. Our results showed that the activation of PPAR‐γ (induced by Rosiglitazone) had little effect on the polarization of macrophage induced by PGE_3_ (Figure S4). However, PPAR‐γ may have anti‐inflammation effect by reducing inflammatory factors produced by macrophage.

## 5 CONCLUSIONS

Our findings reveal a novel mechanism through which eicosanoids can influence tumorigenesis. Specifically, PGE_3_ can influence the macrophage polarization to resolve inflammation and inhibit prostate tumour growth.

## CONFLICT OF INTEREST

The authors declared that they have no conflicts of interest to this work.

## AUTHOR CONTRIBUTIONS

**Jing Cui:** Conceptualization (equal); Data curation (lead); Formal analysis (lead); Investigation (lead); Methodology (lead); Resources (equal); Software (equal); Visualization (equal); Writing‐original draft (lead). **Kai Shan:** Project administration (lead); Software (supporting); Validation (equal); Writing‐review & editing (equal). **qin yang:** Methodology (equal); Visualization (equal). **Yumin Qi:** Formal analysis (equal); Methodology (equal); Resources (equal); Visualization (equal). **Hongyan Qu:** Software (supporting); Visualization (equal). **jiaqi Li:** Data curation (equal). **Rong Wang:** Methodology (supporting). **lingling jia:** Resources (supporting); Software (supporting). **wei chen:** Funding acquisition (supporting); Resources (supporting). **Ninghan Feng:** Conceptualization (equal); Resources (equal); Supervision (equal); Validation (equal); Writing‐review & editing (equal). **Yong Q. Chen:** Conceptualization (lead); Funding acquisition (lead); Project administration (equal); Validation (equal); Visualization (equal); Writing‐original draft (equal); Writing‐review & editing (lead).

## ETHICAL APPROVAL

All procedures were approved by the ethics committee of Jiangnan University (protocol number: JN.NO20191030b048130[289] and JN. No20191115c1081230[306]).

## Supporting information

Fig S1‐S4

## Data Availability

The data used to support this study are available from the corresponding author upon request.
