# Supplementary material for: Prostaglandin E3 attenuates macrophage‐associated inflammation and prostate tumour growth by modulating polarization
Source: J Cell Mol Med. 2021 May 13;25(12):5586–601. doi: 10.1111/jcmm.16570 (PMC8184682; doi:10.1111/jcmm.16570)
Supplement: Supplementary file 1 — Fig S1‐S4 [file JCMM-25-5586-s001.docx]

**Prostaglandin E_3_ attenuates macrophage associated inflammation and prostate tumor growth by modulating polarization**

Jing Cui^1,2^, Kai Shan^1,2^, Qin Yang^1,2^, Yumin Qi^1,2^, Hongyan Qu^1,2^, Jiaqi Li^1,2^, Rong Wang^1,2^, Lingling Jia ^1,2^, Wei Chen^2^, Ninghan Feng^3, *^, Yong Q. Chen^1,2, *^

1 Wuxi School of Medicine, Jiangnan University, Wuxi, China

2 School of Food Science and Technology, Jiangnan University, Wuxi, China

3 Department of Urology, Wuxi No. 2 People’s Hospital, Wuxi, China

* Corresponding author: To whom correspondence should be addressed at [yqchen@jiangnan.edu.cn](mailto:yqchen@jiangnan.edu.cn).

Corresponding author ORCID ID:0000-0003-4747-4708


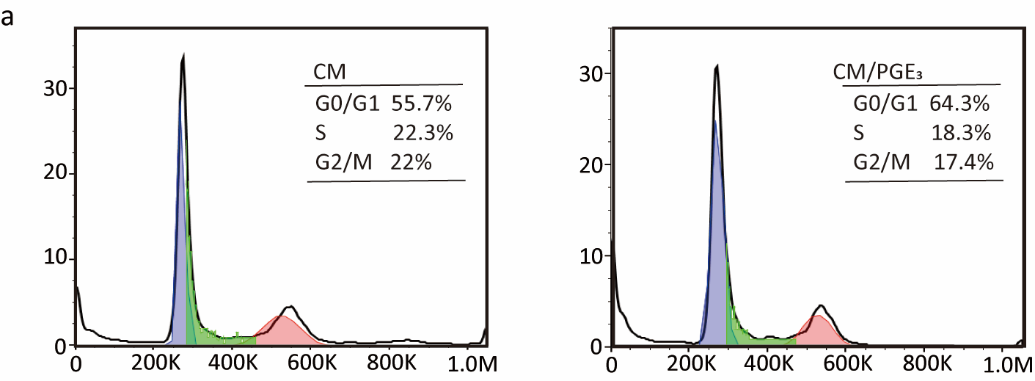


Figure. S1 **PGE_3_ can reverse the proliferation of PC3 cells which was induced by** **conditioned medium.** PC3 cells and THP-1 were co-cultured for 7 ds and treated with or without PGE_3_ (100 nM). The medium was replaced every 2 ds. After 7 ds, the medium was collected as the conditioned medium (CM), or CM/PGE_3_ (PGE_3_ added in the co-culture system). The S/G2 phase of PC3 cells has been downregulated by the CM/PGE3 (35.7%) compared to the CM (44.3%).


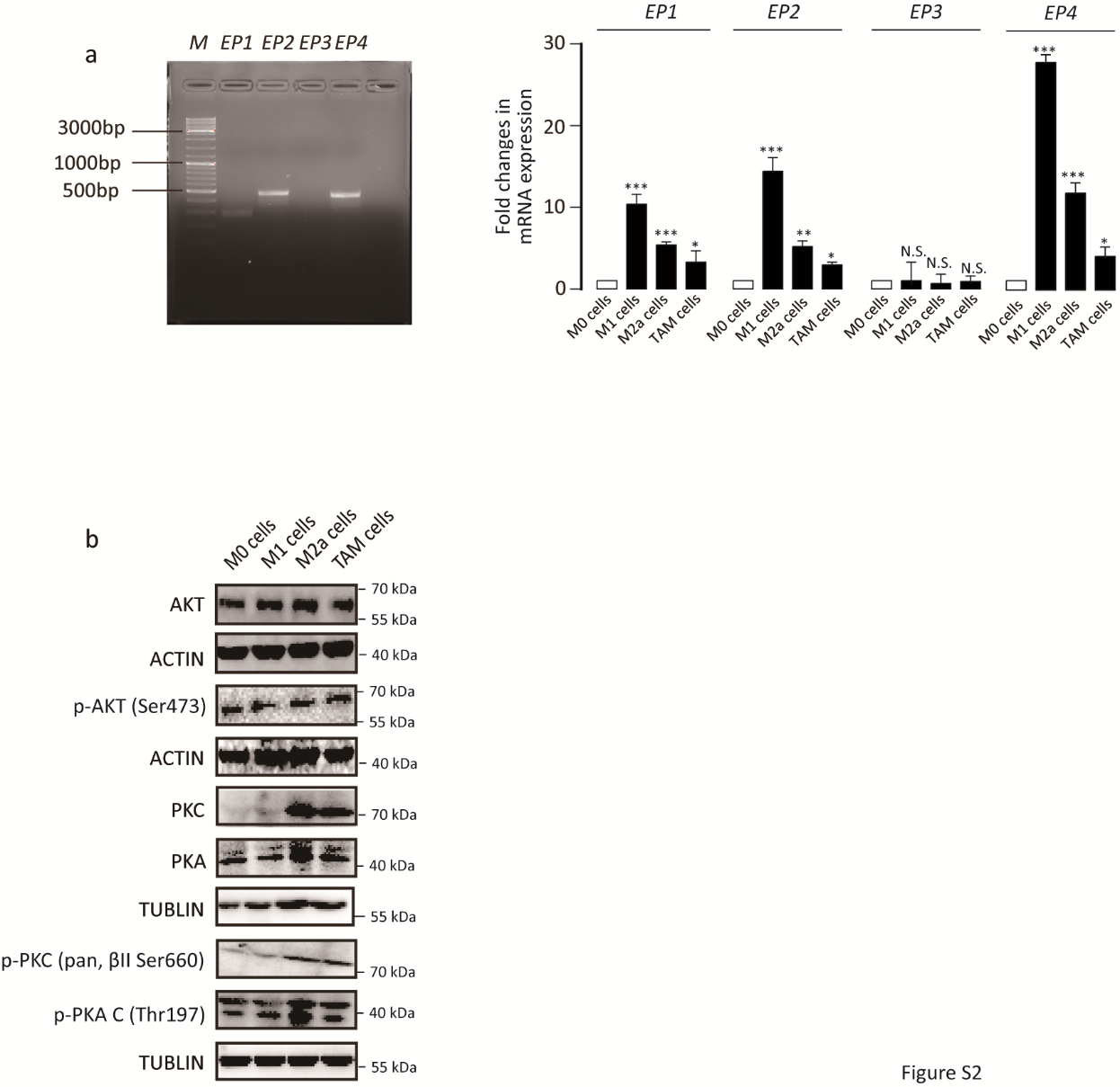


Fig. S2 **Expression of 4 Prostaglandin E receptors and related proteins in THP-1 cells.** (**a)** Expression of EP1, EP2, EP3 and EP4 were detectable by RT-PCR in THP-1 cells, and the RT-PCR products of M0 cells used to run nucleic acid gel. (**b)** The expression of AKT, PKC-β, PKA Cα/β, and their phosphorylation state were detected by western blot and their phosphorylation site are Ser473 for AKT, βII Ser660 for PKC and Thr197 for PKA respectively. Graphs represent means ±SD; N.S.= No significance, *p ≤ 0.05, **p ≤ 0.01, ***p ≤ 0.001


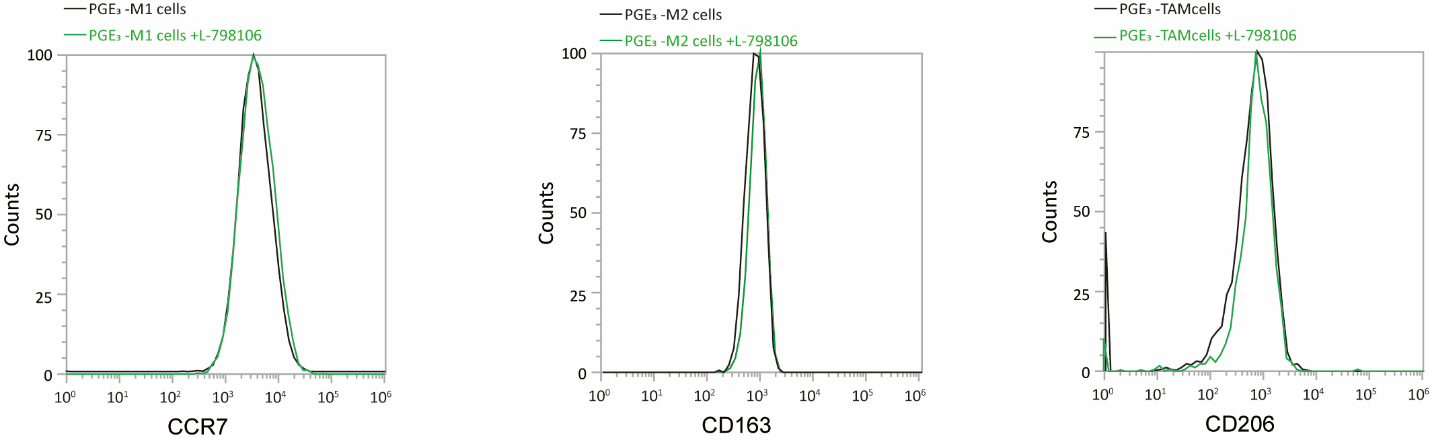


Fig. S3 **Analysis of M1 and M2a markers by flow cytometry after treatment with L-798106 (a EP3 receptor antagonist).**


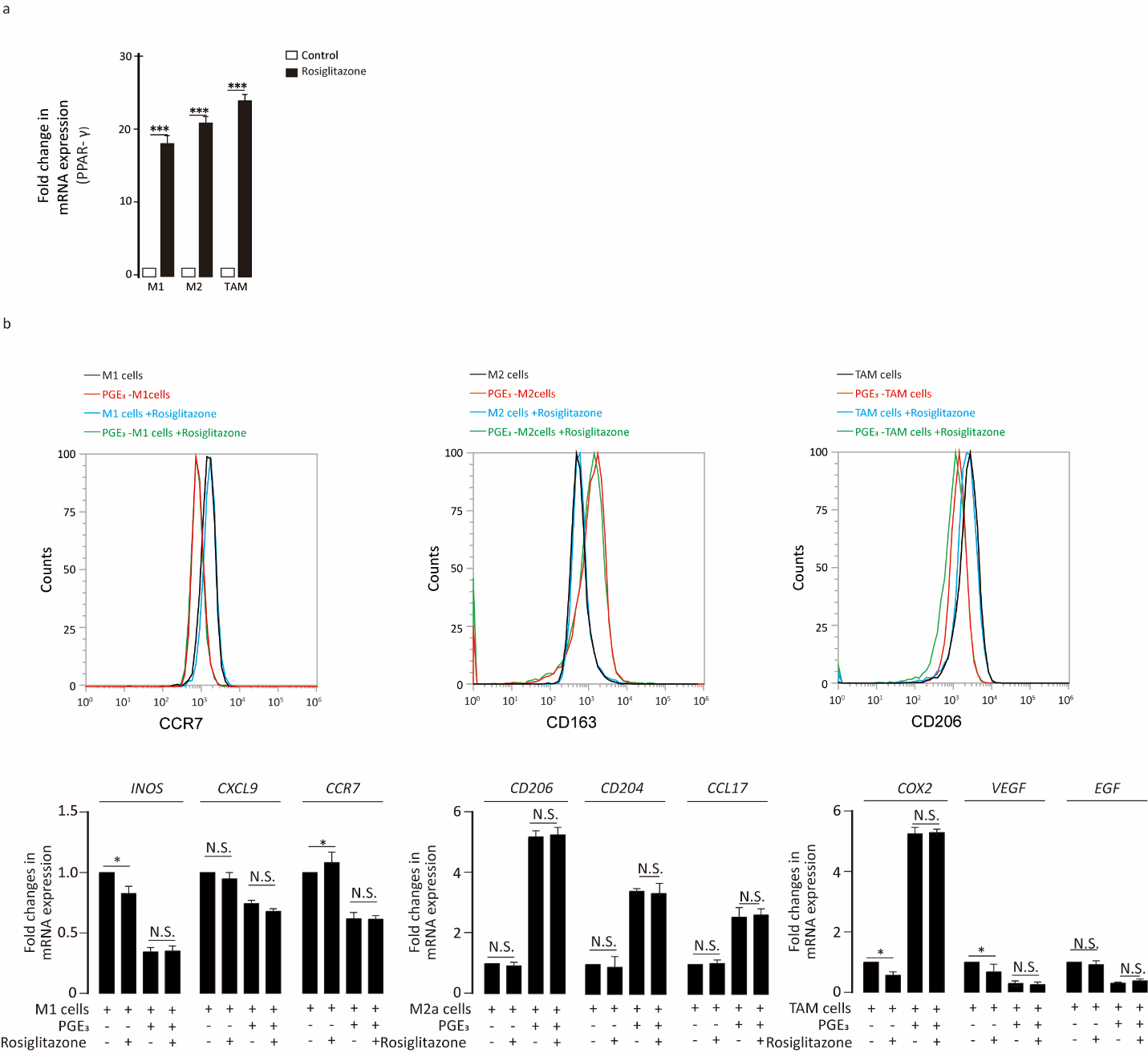


Fig. S4 **Effect of PPAR-γ activation on macrophage polarization. (**a) expression of PPAR-γ after induced by Rosiglitazone. (b) effect of Rosiglitazone on polarization of macrophages with or without PGE_3_ treatment. Graphs represent means ±SD; N.S.= No significance, *p ≤ 0.05.
